# Mapping Obesity Coverage in Florida Counties Using Interactive Web‐Based Mapping Tools to Support Targeted Policy and Intervention Efforts

**DOI:** 10.1155/jobe/8864889

**Published:** 2025-12-30

**Authors:** Justice Moses K. Aheto, Ovie A. Utuama, Getachew A. Dagne

**Affiliations:** ^1^ Department of Biostatistics, School of Public Health, College of Health Sciences, University of Ghana, Accra, Ghana, ug.edu.gh; ^2^ College of Public Health, University of South Florida, Tampa, USA, usf.edu

**Keywords:** adults, Florida, geospatial modeling, obesity, prevalence, risk factors, USA, web-based mapping

## Abstract

**Background/Objectives:**

Obesity is among the most common global public health issues in the 21^st^ century and contributes significantly to cardiovascular morbidity and mortality burden. The success of well‐targeted policies and intervention strategies aimed at addressing obesity depends heavily on understanding the effect of geographical location on obesity and other predictors. The study aim was to quantify county‐level geographical differences in obesity across Florida counties while simultaneously identifying predictors of obesity prevalence.

**Methods:**

This study used the 2019 data from the Florida state‐based telephone surveillance systems, known as the Behavioral Risk Factor Surveillance System (BRFSS) which provides county‐level data on measures of the prevalence of personal health behaviors that are risk factors for morbidity and mortality. The survey collected data on a total sample of 54,260 adults residing in 67 counties of Florida. This study applied Bayesian geospatial models and interactive web‐based mapping approaches to analyze and map county‐level geographical differences in the risk of obesity. The estimated coefficients were presented as log mean with their associated 95% credible intervals (Cr.Is).

**Results:**

The study identified sedentary lifestyle (log mean = 0.023, 95% Cr.I: 0.006, 0.039) as the only risk factor independently associated with increased burden of obesity. The results showed substantial county‐level geographical differences in the predicted obesity prevalence with an overall obesity prevalence of 68.6% with a range of 59.0%–75.7%. Residing in Holmes was associated with the highest burden of obesity. Furthermore, the prevalence was relatively high in Levy, Columbia, Lafayette, Hendry, Bradford, Calhoun, Dixie, Okeechobee, and Gadsden counties.

**Conclusion:**

The substantial county‐level geographical difference in obesity prevalence found is of great importance for sound public health policy and intervention strategies at the local level. The geospatial modeling supported by the web‐based spatial mapping tool employed in this study can help guide the design of geographical prioritization of targeted public health policies and intervention strategies to combat adult obesity and its associated mortality.

## 1. Introduction

With the prevalence of obesity of 40% among adults [1], obesity and its complications were estimated to have contributed more than $140 billion annually to healthcare expenditure in the United States during the early to mid‐2000s [2]. Between 1980 and 2013, there was a reported 28% and 47% increase in the prevalence of obesity among children and adults, respectively [3]. Changing early feeding habits, higher calorie diets, and decreased physical activity among children have been variously identified as drivers of this increase during the period, as overweight children are more likely to become obese adults [4–6]. Furthermore, adult obesity is a risk factor for cardiovascular disease, type 2 diabetes mellitus, and various cancers such as those of the prostate and breast [7–9]. For this reason, obesity underpins the most important causes of morbidity and mortality, and its prevention is central to the health and well‐being of the young and old, as well as men and women.

Sweetened beverages, especially sugar‐sweetened beverages like sodas, energy drinks, sweetened teas, and juices, significantly contribute to the global obesity epidemic attributable to their high sugar content and low nutritional value. Public health policies particularly taxation, marketing restrictions, and labeling have proven to be effective in reducing consumption and, to some extent, improving obesity outcomes. However, lasting impact requires multifaceted strategies, sustained political will, and public support. In the United States, there have been geographic differences in the distribution of obesity, with areas of high prevalence observed in the Midwest and South [10]. While individual level characteristics such as being non‐White, an African American female, of lower income, and of lower educational attainment have been identified as increasing obesity risk [11, 12], the socioeconomic environment in which persons live has not received as much attention. For example, one of the more detailed studies on county characteristics and obesity in the US is based on 2009 data [13]. Another more recent 2016 obesity study, which included crude county obesity rates, did not further characterize the counties to potentially explain their effects on obesity patterns [14].

Florida is the most populous state in the southeastern region of the US, with high racial and ethnic diversity. According to the Bureau of Chronic Disease Prevention of the Department of Health, 34% of Florida adults have healthy weight, while 28% are obese and 36% are overweight [15]. It has been observed that obesity is one of the risk factors for many cancers, cardiovascular disease, and diabetes [16]. It offers a unique opportunity to revisit county characteristics that may be associated with obesity using the most updated data available. There is paucity of data on modeled surfaces of obesity prevalence across counties in Florida using Bayesian modeling and interactive web‐based mapping tools. Therefore, using geospatial modeling under Bayesian framework and interactive web‐based mapping techniques to map obesity in Florida, we aim to characterize the geographical distribution of obesity and evaluate its county‐level predictors for targeted policy and intervention strategies.

## 2. Materials and Methods

### 2.1. Data Source

The data for this study were acquired from the Department of Health State of Florida, Bureau of Community and Health Assessment, Division of Public Health Statistics & Performance Management (DPHSM). The data on the percentages of adults who were overweight or obese (hereafter referred to as obese) by county and associated risk factors came from state‐based telephone surveillance system, known as the Behavioral Risk Factor Surveillance System (BRFSS). The objective of BRFSS is to obtain measures of the prevalence of personal health behaviors that are risk factors for morbidity and mortality [17]. This study used data from a sample size of 54,260 interviews conducted in Florida during the period 2017–2019. The target population for the BRFSS survey consisted of Florida residents who were 18 years and older.

### 2.2. Outcome Variable

The response variable for this study was the percentage of adults, both men and women, who were obese per county in 2019, the most recent and complete data. Being overweight was defined as having a body mass index above 25 but below 30, while being obese was defined as having a body mass index of 30 or greater. Percentages were converted to proportions to allow the response variable to be bounded in the interval (0, 1).

### 2.3. Covariates (Risk Factors)

For explaining the variation in overweight/obese rates, the following county‐level covariates were used in the analysis. These were (i) the percent of Black population to account for racial disparity, (ii) unemployment rate, (iii) education level with 4 categories (percentages of adults with less than high school, high school diploma, associate degree, and bachelor’s degree or higher), (iv) percentage of adults below poverty level, (v) percent of insurance coverage, and (vi) percent of adults who are sedentary. These were the county‐level characteristics whose data were available for analysis and are identified as risk factors for obesity and other health outcomes [18–22].

### 2.4. Statistical Analysis

The study utilized Bayesian geospatial models to quantify and map the risk of obesity among adults across all 67 counties in Florida while simultaneously adjusting for its associated county‐level risk factors.

#### 2.4.1. Model Formulation

**Y**_**i**_ was defined as the observed proportion of obesity prevalence in county *i,* and **X**_**i**_ a vector of regressors (i.e., risk factors or covariates). It was assumed that *Y*_*i*_ were conditionally and independently beta distributed with *E*(*Y*) = *μ* and precision parameter *ϕ* presented as

(1) Yiβ,ϕ∼Betaμi, ϕi,i=1267,,…,.

Following the Besag–York–Mollié (BYM) model [16], the beta spatial bounded distribution model was presented as

(2) gμi=β0+dxi′β+wi+vi,

where *g*(.): (0, 1) is a link function strictly increasing, here the logit link.

The model in equation (2) could also be presented as

(3) log  μi1−μi=β0+dxi′β+wi+vi,

where *β*_0_ defined the overall mean (i.e., intercept), *β* is a vector of regression coefficients associated with the considered risk factors, *w*_*i*_ is the structured spatial random effect vector associated with county *i*, which allowed for the county‐level spatial dependency, and *v*_*i*_ is the unstructured spatial effect for nonspatial heterogeneity distributed as $v_{i}∼N\left(0,\;\sigma_{v}^{2}\right)$. The term *w*_*i*_ was modeled using the intrinsic conditional autoregressive (ICAR) distribution given as $w_{i}\left.\;\right|\;w_{-i}\;∼\;N\left(\overset{¯}{w}\delta_{i},\;\sigma_{w}^{2}/\left(n\delta_{i}\right)\right)$. *δ*_*i*_ represented the neighborhood of the *i*th county, $n_{\delta_{i}}$ was the number of counties in the *i*th neighborhood, and $\overset{¯}{w}\delta_{i}$ was the mean of the neighboring *w*_*i*_ values. Detailed description of the methods is available elsewhere [10].

The model was implemented under the Bayesian framework through the novel and efficient recommended integrated nested Laplace approximation (INLA) with stochastic partial differential equation (SPDE) strategy [23, 24]. The default noninformative priors in the R‐INLA package were used to complete the model because the study lacks reliable priors about the model parameters, and all the analyses were carried out in the R‐INLA package [25, 26]. To declare statistical significance, 95% Bayesian credible intervals (Cr.Is) were employed. A cross‐validation procedure was employed to assess predictive power of the fitted model since the goal of the study is the prediction and accuracy of the model’s predictive performance.

The study produced exceedance probabilities using the predicted obesity prevalence (*p*) for each county. The goal was to identify counties where the predicted prevalence exceeded a given threshold, in this case the 70^th^ (i.e., Pr (*p* > 70%)) and 75^th^ (i.e., Pr (*p* > 75%)) percentiles. These thresholds could be reduced or increased depending on the needs of policymakers and program managers and the available resources to support such thresholds for targeted interventions.

To support sound programmatic policy and intervention strategies amidst limited public health resources, interactive web‐based spatial mapping tools were developed to improve the visualization and identification of high‐risk obesity burden as well as low‐risk burden counties in Florida. For each county, the study simultaneously mapped the name of the county, the observed and predicted obesity prevalence, the lower and upper 95% Bayesian Cr.Is for the predicted prevalence, and significant risk factors (i.e., sedentary (%)) using an interactive web‐based visualization tool. The *leaflet*, *sp*, and *rgdal* packages in R Version 4.2.3 and RStudio were used to develop the interactive web‐based spatial mapping tool.

## 3. Results

### 3.1. Predictors of Obesity in the Spatial Model

**Figure 1:**
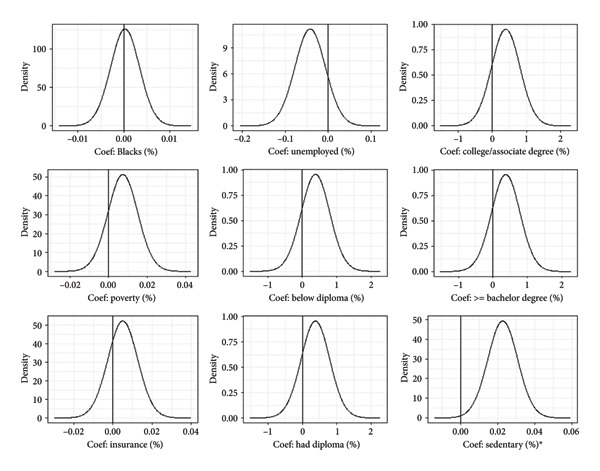
Plots of posterior distribution of the model parameters in the beta spatial model. Note: ∗ denotes significant risk factor. The solid lines represent the reference value of zero (0).

The crude/observed obesity prevalence was 68.2% with a range of 54.6%–78.5%. Our study identified sedentary lifestyle (i.e., physical inactivity) to be the only risk factor independently associated with increased log‐odds (log mean = 0.023, 95% CI: 0.006, 0.039) of obesity in the spatial model. Unemployment was associated with decreased log‐odds of obesity, albeit statistically insignificant. Percent of Blacks, college/associate degree, below diploma, diploma, and bachelor’s degree holders, poverty, and insurance increased the log‐odds of obesity, albeit statistically insignificant (Figure 1).

### 3.2. Spatial Variation of Obesity

**Figure 2:**
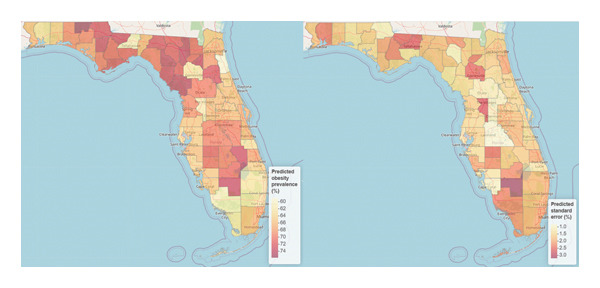
Maps of the predicted obesity prevalence (left panel) and its associated standard deviation (right panel) in Florida counties. Note: the interactive web‐based versions of this map named Map 1 (left panel) and Map 2 (right panel) are available at the link online.

The results showed substantial county‐level geographical differences in the predicted obesity prevalence in Florida with an overall obesity prevalence of 68.6% with a range of 59.0%–75.7%. The highest prevalence was recorded in Holmes (prevalence (*p*): 75.7%, 95% Cr.I: 72.5%–78.8%). The risk of obesity was found to be higher in Levy (*p*: 74.4%, Cr.I: 71.4%–77.2%), Columbia (*p*: 74.0%, Cr.I: 70.8%–77.2%), Lafayette (*p*: 73.9%, Cr.I: 70.3%–77.4%), Hendry (*p*: 73.9%, Cr.I: 67.4%–79.9%), Bradford (*p*: 73.8%, Cr.I: 70.5%–76.8%), Calhoun (*p*: 73.5%, Cr.I: 69.2%–77.6%), Dixie (*p*: 73.3%, Cr.I: 70.5%–76.1%), Okeechobee (*p*: 73.3%, Cr.I: 70.0%–76.4%), and Gadsden (*p*: 73.3%, Cr.I: 68.1%–78.2%). Other relatively high‐risk counties include Jackson (*p*: 72.9%, Cr.I: 70.7%–75.0%), Madison (*p*: 72.8%, Cr.I: 69.1%–76.4%), Putnam (*p*: 72.5%, Cr.I: 69.6%–75.4%), and Suwannee (*p*: 72.3%, Cr.I: 70.1%–74.3%) counties. The lowest prevalence was observed in St. Johns County (*p*: 59.0%, Cr.I: 54.9%–63.0%) which is the only county that recorded a predicted prevalence below 60% in Florida (see Figure 2 and supporting Table S1). The predicted standard deviation associated with our predicted prevalence ranged from 0.8 in Polk to 3.2% in Hendry with an overall mean and median standard deviation of 1.8% and 1.7% respectively, suggesting that the uncertainty associated with our prediction was low (Figure 2). The interactive web‐based spatial map versions of Figure 2 named Map 1 (left panel) and Map 2 (right panel) are available at the link https://drive.google.com/drive/folders/1lPRGgW1tSZwD74pqAFu9BwXEG_J7U0wO.

**Figure 3:**
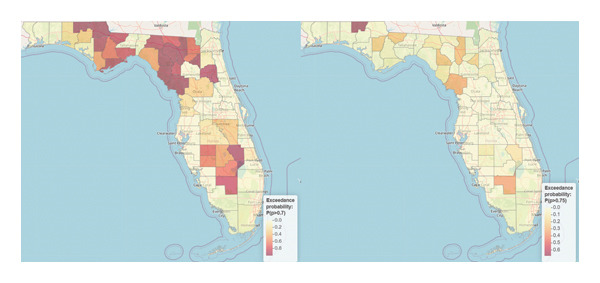
Maps of the exceedance probability for 70^th^ (left panel) and 75^th^ (right panel) percentiles in Florida counties. Note: the interactive web‐based versions of this map named Map 3 (left panel) and Map 4 (right panel) are available at the link online.

The exceedance probability map for 70^th^ percentile identified Holmes, Levy, Columbia, Lafayette, Bradford, Calhoun, Dixie, Okeechobee, Gadsden, Jackson, Madison, Putnam, Franklin, and Suwannee to be Florida counties at the highest risk of obesity. For the 75^th^ percentile, only Holmes County was identified to be at the highest risk of obesity (Figure 3). The interactive web‐based spatial map versions of Figure 3 named Map 3 (left panel) and Map 4 (right panel) are available at the link https://drive.google.com/drive/folders/1lPRGgW1tSZwD74pqAFu9BwXEG_J7U0wO.

### 3.3. Model Validation

**Figure 4:**
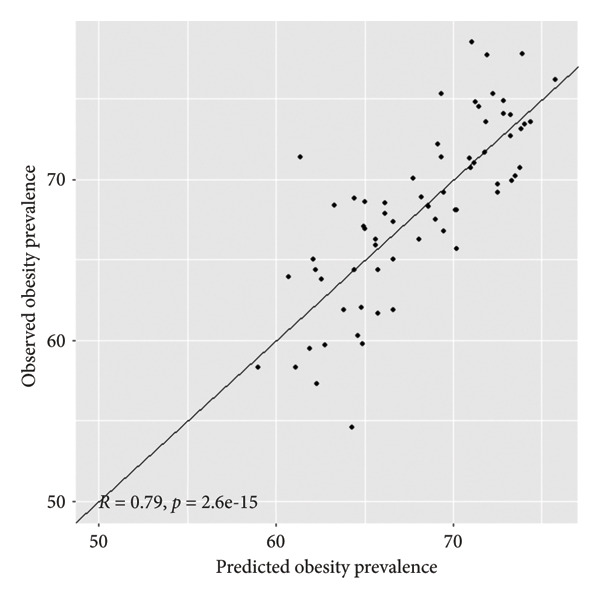
Model validation plot for the Bayesian beta spatial model for predicting obesity prevalence among adults in Florida counties.

The results presented in Figure 4 showed a high and significant correlation between the observed and predicted obesity prevalence in this population of adults in Florida counties. This strongly suggests that the model performs very well in predicting obesity prevalence spatially across counties in Florida.

## 4. Discussion

In this study, county‐level geographical difference in obesity prevalence among adults was investigated. The results revealed three key findings. First, residing in the northwestern counties such as Calhoun, Franklin, and Gadsden was associated with the highest burden of obesity, although there were pockets of high burden in the northeastern counties of Columbia, Lafayette, Bradford, and Dixie, in the central western county of Levy, in the central eastern county of Okeechobee, and in the southwestern county of Hendry. Second, the percentage of persons with a sedentary lifestyle was the only modifiable risk factor independently associated with an increased burden of obesity in Florida. Third, the study developed exceedance probabilities for two (2) separate thresholds of prevalence, both of which identified Holmes County to be at the highest risk of obesity. Taken together, the findings of the present study suggest that a sedentary lifestyle is likely a primary driver of obesity in the northwestern region of Florida, in which Holmes County is a part. Furthermore, given that Florida has one of the oldest populations in the United States, its northwest may represent an unusual confluence of high comorbidities, mortality, and healthcare expenditure, making the region an ideal location to pilot obesity‐related interventions.

The main findings of the present study are consistent with other geospatial studies that relied on BRFSS estimates and have also demonstrated the high prevalence of obesity in the northwest of Florida [21, 22]. In one of these studies comparing obesity estimates from 2012 through 2016 of de‐identified electronic health records (EHRs) across Florida with 2013 BRFSS weighted state estimates, not only was obesity concentrated in the northwest by both methods, but also in the southeast with the highest prevalence among the 40 to 59 age group [14]. Although the pattern of obesity distribution was consistent across the state, EHR obesity estimates by age, sex, and race were consistently larger than the 2013 BRFSS estimates (e.g., 41.6% vs. 31.7% obesity prevalence among 40–59 age group). This may have been due to the tendency of hospitals pooling a sicker portion of the population who were more likely to be comorbidly obese and BRFSS data collection methodology, which relies on respondents’ recall, tending to produce dated or inaccurate weight estimates. For these reasons, obesity among adults in Florida may be a larger issue than previously acknowledged.

In comparing counties with at least a 70% rate of obesity with St. Johns, the only county with BRFSS and predicted obesity prevalence lower than 60%, we observed that the former differed from St. Johns in their high double‐digit prevalence of poverty and lower proportion of adults with at least a bachelor’s degree. Our findings suggest that low poverty rates, as exemplified by St. Johns’ 9%, tended to be protective of obesity in counties and this effect may have been modified by high educational attainment. We observed this potential effect modification in Alachua and Leon counties, in that despite poverty rates above 20% with almost half of their populations estimated to have had at least a bachelor’s degree, county obesity rates predicted by our model were in the low 60% in addition to having lower sedentary lifestyles among 22% and 19% of residents, respectively. As has been reported elsewhere, the present study implies that obesity is a by‐product of the interplay of both adverse and protective sociodemographic factors [27]. It is worth noting that neither county poverty nor education variables were statistically significant, and this differs from results of an obesity study of more than 3000 counties across the US [21]. In that study, educational level, poverty, healthcare context, and sedentary lifestyle were statistically significant, regionally. It is likely the present study was limited by its small county numbers and therefore underpowered to detect differences in poverty and education.

This study developed interactive web‐based spatial mapping tools and produced obesity risk maps superimposed with individual county names, observed obesity prevalence, predicted obesity prevalence, and associated 95% lower and upper bound Bayesian Cr.Is and identified significant risk factors. By placing a cursor on any county polygon in the interactive web‐based maps for the predicted prevalence, the tool automatically provided the user with parameters/details about that county for improved visualization. The color coding provided alerts to the user about the level of obesity burden in that county with the red color representing higher burden obesity counties in Florida. This standalone but effective tool could support policymakers and program managers in charge of disease prevention, control, surveillance, and elimination efforts to ensure that the implementation of policies, interventions, and programs is targeted at the counties in most need. To the best knowledge of the authors, this study is the first study that developed an interactive web‐based spatial mapping tool which simultaneously superimposed several model parameters in each county to support intervention efforts aimed at reducing the burden of obesity in Florida.

This study is cross‐sectional in nature and so cannot be used to draw causative inference. The study did not consider all potential county‐level predictors of obesity due to the limited number of variables of importance available in the survey data, nor can county‐level associations with obesity (or lack thereof) be deduced to reflect equivalence at the individual level. Given that Florida has an older population with larger ethnic diversity, our findings may not be generalizable to younger less diverse populations. This implies that obesity interventions in the state would benefit from not only the common school‐based programs but also community‐based ones which equally target older, racially, and ethnically different populations.

## 5. Conclusion

In summary, the study found substantial county‐level geographical differences in the burden of obesity across all 67 counties in Florida and identified sedentary lifestyle as a key predictor of obesity in this population. The higher burden obesity risk counties identified should be urgently targeted with additional but effective obesity control programs to reduce the risk of obesity morbidity and its associated mortality. Geospatial modeling and interactive web‐based mapping could serve as a critical tool to support policymakers and program managers for obesity planning, prevention, and control efforts in Florida. As part of an overall strategy aimed at addressing the problem of obesity morbidity and mortality, there is a need to search for as‐yet unidentified risk factors (e.g., culture and religion) not considered in this study using other methodologies such as latent factor analysis design and carry out individual‐level studies to ascertain the interplay of known social determinants of health and obesity, as well as expand obesity programs beyond schools and into communities.

**Nomenclature**
BRFSS: Behavioral Risk Factor Surveillance System
DPHSM: Department of Health State of Florida, Bureau of Community and Health Assessment, Division of Public Health Statistics & Performance Management

## Ethics Statement

This is not applicable to this study because the study is a secondary data analysis which utilized publicly and freely available data for research and academic purposes. Also, the publicly available data are aggregated at the county level and de‐identified, making it impossible to identify or contact the individual respondents who participated in the main survey.

## Disclosure

All authors have read and approved the final manuscript.

## Conflicts of Interest

The authors declare no conflicts of interest.

## Author Contributions

Justice Moses K. Aheto and Getachew A. Dagne developed the concept. Justice Moses K. Aheto and Getachew A. Dagne secured the data. Justice Moses K. Aheto analyzed the data and wrote the first draft manuscript. Justice Moses K. Aheto, Ovie A. Utuama, and Getachew A. Dagne contributed to the writing and reviewing of the various sections of the manuscript. All authors reviewed the final version of the manuscript before submission.

## Funding

The authors declare that this study received no funding.

## Supporting Information

To put our spatial results in context, we have provided obesity prevalence by county and county characteristics such as race, employment status, education, poverty levels, insurance, sedentary life status, and observed and predicted obesity prevalence among adults in Florida in Supporting Table S1.

## Supporting information


**Supporting Information** Additional supporting information can be found online in the Supporting Information section.

## Data Availability

The data used for our analyses are freely available at https://www.flhealthcharts.gov/ChartsReports/rdPage.aspx?rdReport%3d;BrfssCounty.DataViewer%26;bid%3d;77.
