# Supplementary material for: Mapping Obesity Coverage in Florida Counties Using Interactive Web‐Based Mapping Tools to Support Targeted Policy and Intervention Efforts
Source: J Obes. 2025 Dec 30;2025:8864889. doi: 10.1155/jobe/8864889 (PMC12767032; doi:10.1155/jobe/8864889)
Supplement: Supplementary file 1 — Supporting Information Additional supporting information can be found online in the Supporting Information section. [file JOBE-2025-8864889-s001.docx]

**Mapping obesity coverage in Florida counties using interactive web-based mapping tools to support targeted policy and intervention efforts**

Justice Moses K. Aheto, Ovie A. Utuama, Getachew A. Dagne

**Supplementary Table**

Table S1 Obesity prevalence by county characteristics among adults in Florida, 2015-2019

| County | %  black | %  unemployment | %  < high school diploma | %  high school diploma only | %  some college | %  ≥ bachelor's degree | %  poverty | %  insured | %  sedentary | %  obesity (BRFSS) | %  obesity (predicted) |
| --- | --- | --- | --- | --- | --- | --- | --- | --- | --- | --- | --- |
| Alachua | 20.6 | 4.3 | 7.4 | 21.2 | 28.1 | 43.3 | 24.2 | 89.7 | 21.8 | 57.3 | 62.3 |
| Baker | 14 | 4.9 | 14.3 | 42.6 | 29.9 | 13.2 | 18.5 | 86.7 | 34.9 | 70.7 | 71.0 |
| Bay | 11.7 | 4.8 | 9.3 | 31.0 | 35.9 | 23.7 | 15.6 | 82.1 | 29.7 | 66.3 | 68.5 |
| Bradford | 20.1 | 4.2 | 20.4 | 40.4 | 29.4 | 9.9 | 19.3 | 83.8 | 34.9 | 70.7 | 73.8 |
| Brevard | 10.7 | 5.1 | 7.8 | 28.3 | 33.8 | 30.2 | 14.0 | 86.2 | 27.9 | 67.4 | 66.6 |
| Broward | 30.1 | 4.5 | 11.0 | 27.3 | 29.2 | 32.4 | 14.4 | 85.6 | 24.1 | 65.0 | 62.1 |
| Calhoun | 13.2 | 5.7 | 23.1 | 41.6 | 25.8 | 9.5 | 13.1 | 84.3 | 38.7 | 70.2 | 73.5 |
| Charlotte | 6 | 5.3 | 9.4 | 34.6 | 32.6 | 23.4 | 12.4 | 87.9 | 25.8 | 66.9 | 65.0 |
| Citrus | 3.1 | 6.7 | 11.6 | 37.2 | 32.9 | 18.3 | 17.7 | 87.7 | 32.3 | 67.5 | 69.0 |
| Clay | 12.3 | 4.5 | 8.2 | 31.0 | 35.8 | 24.9 | 10.2 | 86.1 | 31.6 | 68.9 | 68.2 |
| Collier | 7.4 | 4.7 | 12.9 | 25.8 | 24.9 | 36.4 | 13.2 | 75.5 | 25.4 | 63.9 | 60.7 |
| Columbia | 18.5 | 4.9 | 13.9 | 36.7 | 34.6 | 14.9 | 19.4 | 83.9 | 36.7 | 73.4 | 74.0 |
| DeSoto | 12.6 | 5.3 | 26.5 | 43.8 | 18.2 | 11.5 | 29.9 | 82 | 32.3 | 71.3 | 70.9 |
| Dixie | 9.8 | 5.4 | 21.6 | 43.3 | 24.8 | 10.4 | 21.5 | 82.2 | 38.1 | 69.9 | 73.3 |
| Duval | 30.6 | 4.9 | 10.2 | 27.8 | 31.9 | 30.0 | 16.6 | 83.8 | 29.4 | 68.5 | 66.1 |
| Escambia | 23.2 | 4.9 | 9.5 | 27.2 | 36.7 | 26.5 | 14.7 | 83 | 27 | 67.9 | 66.2 |
| Flagler | 10.7 | 5.5 | 7.8 | 34.3 | 32.8 | 25.1 | 13.2 | 91.1 | 24.7 | 64.4 | 64.4 |
| Franklin | 12.4 | 4.3 | 19.3 | 34.6 | 27.3 | 18.8 | 20.7 | 87.9 | 34.8 | 69.2 | 72.5 |
| Gadsden | 55.8 | 6.2 | 20.2 | 36.2 | 27.4 | 16.2 | 25.6 | 84.3 | 38.9 | 72.7 | 73.3 |
| Gilchrist | 5.6 | 5.0 | 14.6 | 42.1 | 29.9 | 13.4 | 23.8 | 81.8 | 34.5 | 77.7 | 71.9 |
| Glades | 13.8 | 6.2 | 25.5 | 38.2 | 25.1 | 11.2 | 20.7 | 88.2 | 36.8 | 78.5 | 71.0 |
| Gulf | 17 | 4.6 | 14.5 | 35.8 | 30.4 | 19.2 | 15.3 | 86 | 31.4 | 68.1 | 70.2 |
| Hamilton | 32.8 | 5.6 | 27.0 | 45.4 | 19.8 | 7.9 | 27.0 | 78.4 | 33.1 | 73.6 | 71.9 |
| Hardee | 7.5 | 6.6 | 22.8 | 49.1 | 17.8 | 10.2 | 26.4 | 81.7 | 42.7 | 71.7 | 71.8 |
| Hendry | 12.1 | 8.5 | 33.7 | 33.7 | 24.3 | 8.3 | 26.3 | 82.1 | 42 | 73.1 | 73.9 |
| Hernando | 6 | 6.0 | 12.1 | 35.2 | 34.2 | 18.4 | 15.6 | 85.3 | 31.8 | 68.3 | 68.6 |
| Highlands | 10.5 | 6.5 | 14.3 | 38.8 | 29.8 | 17.2 | 19.4 | 81.5 | 34 | 65.7 | 70.2 |
| Hillsborough | 17.8 | 4.4 | 11.3 | 27.1 | 28.0 | 33.5 | 16.4 | 83 | 27.4 | 68.8 | 64.5 |
| Holmes | 6.7 | 5.4 | 21.3 | 40.7 | 27.3 | 10.7 | 26.0 | 81.7 | 40 | 76.2 | 75.7 |
| Indian River | 9.5 | 6.0 | 10.6 | 30.4 | 29.8 | 29.1 | 14.1 | 84.7 | 26.7 | 59.7 | 62.8 |
| Jackson | 26.9 | 5.2 | 19.5 | 38.4 | 29.5 | 12.6 | 22.9 | 82.6 | 35.7 | 74.1 | 72.9 |
| Jefferson | 34.3 | 5.2 | 18.0 | 34.7 | 25.0 | 22.3 | 15.8 | 87.1 | 29.6 | 65.0 | 66.7 |
| Lafayette | 13.5 | 4.3 | 24.7 | 38.9 | 24.6 | 11.8 | 18.0 | 82.6 | 39.5 | 77.8 | 73.9 |
| Lake | 11.3 | 4.8 | 10.5 | 32.1 | 33.4 | 24.0 | 13.5 | 83.5 | 26.4 | 64.4 | 65.8 |
| Lee | 9.1 | 4.6 | 11.6 | 31.0 | 29.2 | 28.2 | 15.5 | 84.2 | 26.2 | 62.0 | 64.8 |
| Leon | 31.7 | 4.5 | 6.5 | 19.0 | 28.3 | 46.2 | 21.3 | 91.1 | 19.3 | 58.3 | 61.1 |
| Levy | 9.4 | 5.2 | 15.1 | 41.1 | 31.6 | 12.2 | 22.2 | 82.7 | 40.2 | 73.6 | 74.4 |
| Liberty | 19.6 | 5.1 | 18.6 | 45.8 | 21.2 | 14.4 | 16.9 | 81.5 | 36.5 | 71.0 | 71.2 |
| Madison | 37.8 | 5.2 | 19.0 | 39.7 | 27.3 | 14.0 | 28.5 | 88.7 | 34.7 | 74.9 | 72.8 |
| Manatee | 9.1 | 4.5 | 10.3 | 30.1 | 29.9 | 29.8 | 14.3 | 86.3 | 26.1 | 61.7 | 65.8 |
| Marion | 13.5 | 5.8 | 12.4 | 36.9 | 30.6 | 20.1 | 18.2 | 85.5 | 32.3 | 66.8 | 69.5 |
| Martin | 5.7 | 4.8 | 9.0 | 24.9 | 32.0 | 34.1 | 11.8 | 86.1 | 26.6 | 54.6 | 64.3 |
| Miami Dade | 17.7 | 5.3 | 18.6 | 27.3 | 24.4 | 29.8 | 19.9 | 76.7 | 30.7 | 61.9 | 66.6 |
| Monroe | 6.9 | 3.2 | 8.6 | 26.7 | 30.3 | 34.4 | 13.0 | 74.8 | 23.2 | 64.4 | 62.2 |
| Nassau | 6 | 4.5 | 8.7 | 31.7 | 30.0 | 29.6 | 12.7 | 90.4 | 25.8 | 68.6 | 65.1 |
| Okaloosa | 10.3 | 4.0 | 7.6 | 24.7 | 36.5 | 31.1 | 12.0 | 79.8 | 27.2 | 67.1 | 65.0 |
| Okeechobee | 9 | 5.1 | 22.8 | 39.5 | 26.3 | 11.3 | 25.3 | 83.8 | 37.7 | 74.0 | 73.3 |
| Orange | 22.7 | 4.3 | 11.5 | 24.4 | 29.6 | 34.6 | 17.3 | 79.7 | 27 | 65.9 | 65.6 |
| Osceola | 13.9 | 4.9 | 13.3 | 31.3 | 33.7 | 21.8 | 19.5 | 77.1 | 29 | 75.3 | 69.3 |
| Palm Beach | 19.7 | 4.7 | 11.5 | 24.0 | 27.8 | 36.7 | 13.9 | 85.8 | 25.6 | 59.5 | 61.9 |
| Pasco | 6.5 | 5.1 | 10.5 | 33.2 | 32.2 | 24.0 | 13.6 | 81.8 | 27.5 | 59.8 | 64.9 |
| Pinellas | 11.1 | 4.3 | 8.7 | 28.0 | 31.6 | 31.7 | 14.1 | 85.2 | 22 | 63.8 | 62.6 |
| Polk | 16.1 | 5.5 | 15.0 | 34.7 | 30.1 | 20.2 | 17.7 | 84.2 | 31.7 | 71.4 | 69.3 |
| Putnam | 16.4 | 6.3 | 18.2 | 41.2 | 28.3 | 12.3 | 27.0 | 86.2 | 35.9 | 69.7 | 72.5 |
| Santa Rosa | 6.5 | 5.8 | 5.2 | 21.4 | 28.7 | 44.7 | 12.3 | 89.1 | 29.4 | 68.4 | 63.3 |
| Sarasota | 4.8 | 4.5 | 13.7 | 33.2 | 31.5 | 21.6 | 11.0 | 88.6 | 22 | 61.9 | 63.9 |
| Seminole | 12.9 | 4.5 | 8.7 | 26.8 | 36.8 | 27.7 | 12.1 | 87.2 | 22.6 | 60.3 | 64.6 |
| St. Johns | 5.5 | 6.3 | 6.9 | 28.9 | 28.8 | 35.4 | 9.0 | 91.3 | 22.2 | 58.3 | 59.0 |
| St. Lucie | 21 | 3.8 | 5.7 | 21.1 | 33.6 | 39.6 | 17.9 | 85.5 | 30.3 | 70.1 | 67.8 |
| Sumter | 7.5 | 7.1 | 7.9 | 30.4 | 30.0 | 31.8 | 9.9 | 94.9 | 21.5 | 71.4 | 61.4 |
| Suwannee | 12.8 | 4.9 | 17.7 | 38.9 | 27.9 | 15.5 | 22.7 | 79.5 | 36.2 | 75.3 | 72.3 |
| Taylor | 19.6 | 5.4 | 21.2 | 43.8 | 26.6 | 8.3 | 14.8 | 86.7 | 32.9 | 68.1 | 70.2 |
| Union | 22.7 | 4.3 | 23.5 | 37.4 | 29.9 | 9.2 | 22.4 | 84.7 | 26.4 | 74.8 | 71.2 |
| Volusia | 11.4 | 5.0 | 9.5 | 31.8 | 35.0 | 23.7 | 16.7 | 82.5 | 25.1 | 66.3 | 65.6 |
| Wakulla | 13.1 | 4.1 | 12.3 | 36.2 | 33.2 | 18.3 | 14.3 | 86.7 | 33.8 | 74.5 | 71.5 |
| Walton | 5.2 | 4.3 | 10.9 | 28.5 | 32.1 | 28.5 | 17.4 | 85 | 31.4 | 72.2 | 69.1 |
| Washington | 14.9 | 5.1 | 18.7 | 40.1 | 28.6 | 12.5 | 20.5 | 81.6 | 33.6 | 69.2 | 69.5 |
